# Targeting host ferroptosis in cancer cachexia: Tumor-immune triggers, tissue wasting, and the therapeutic paradox

**DOI:** 10.1016/j.isci.2026.117764

**Published:** 2026-10-05

**Authors:** Yan Li, Rui Chen, Shengguang Ding

**Affiliations:** 1 Department of Cardiovascular Surgery, Southeast University Affiliated Nantong First People’s Hospital, Nantong 226001, P.R. China; 2 Department of Pathology, Nantong University, Nantong 226001, P.R. China; 3 Department of Thoracic Surgery, Southeast University Affiliated Nantong First People’s Hospital, Nantong 226001, P.R. China

**Keywords:** cancer cachexia, ferroptosis, skeletal muscle wasting, adipose tissue loss, iron metabolism, lipid peroxidation

## Abstract

Cancer cachexia is a systemic wasting syndrome driven by tumor burden, chronic inflammation, disrupted nutrient handling, and neuroendocrine stress. Supportive nutrition, exercise, and symptom management rarely restore lean mass, function, or treatment tolerance once wasting is established. Ferroptosis, iron-dependent cell death caused by phospholipid peroxidation, may connect inflammatory iron redistribution, lipid remodeling, mitochondrial stress, and impaired antioxidant defenses. This review critically evaluates ferroptosis in cachexia from a tumor-host pharmacology perspective, distinguishing causal evidence from associated oxidative signatures. We highlight two experimentally supported mechanisms: neutrophil-derived lipocalin-2 in lung cancer cachexia and tumor exosomal miR-203a-3p in pancreatic cancer-associated muscle wasting. We also examine the paradox that tumor ferroptosis induction may weaken host-tissue resistance and worsen muscle and adipose loss. We propose a “split-ferroptosis” strategy combining tumor sensitization with host-protective iron modulation, lipid peroxide scavenging, endogenous defense reinforcement, and biomarker-guided patient selection to expand the tumor-host therapeutic index.

## Graphical abstract

**Figure:**
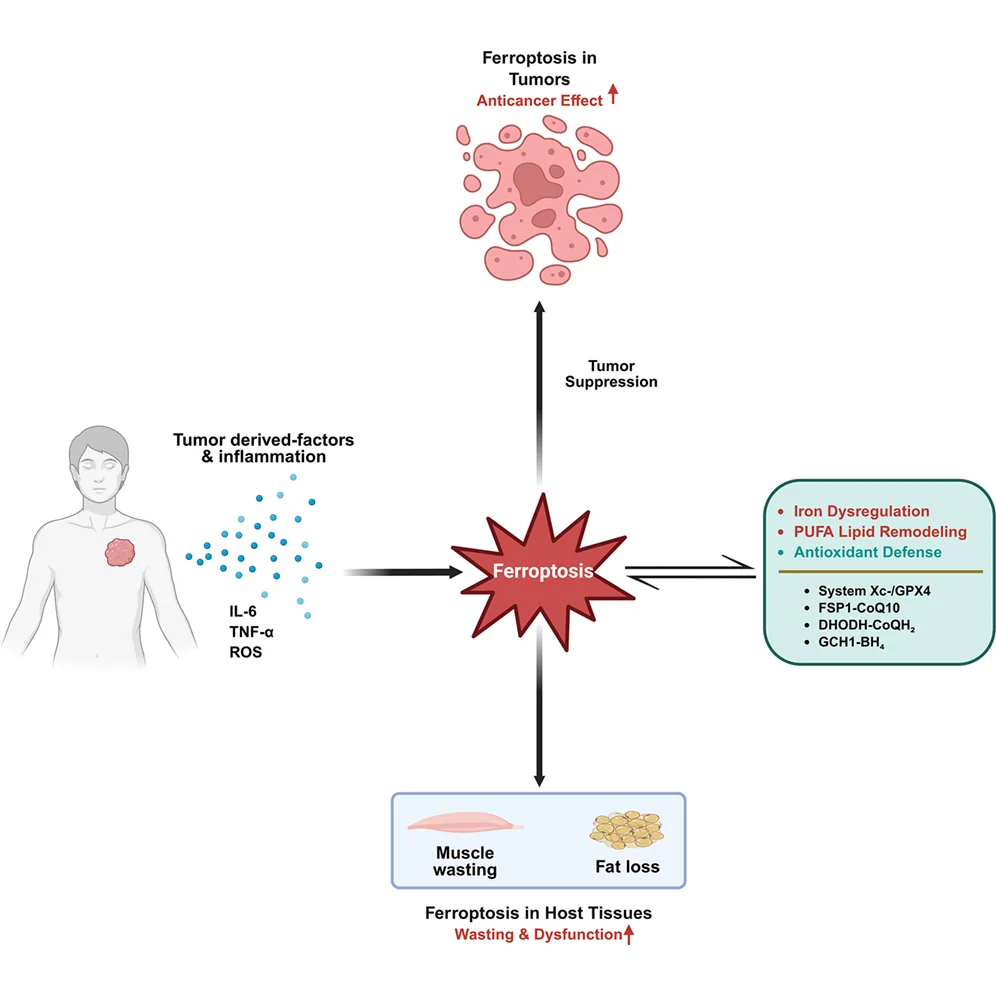

## Teaser

Therapeutics; Microenvironment; Human metabolism; Cancer

## Introduction

Cancer cachexia (CC) is a multifactorial syndrome defined by ongoing skeletal muscle loss, with or without adipose tissue depletion, that cannot be fully reversed by conventional nutritional support. It compromises treatment tolerance, physical function, quality of life, and survival in patients with advanced malignancies.^1^^,^^2^^,^^3^ Contemporary guidelines emphasize early screening, nutrition, exercise, symptom control, and selected appetite- or weight-directed agents, but they also recognize the absence of a broadly accepted disease-modifying pharmacotherapy that reliably restores muscle function or improves survival.^4^^,^^5^^,^^6^ Recent GDF15-targeted ponsegromab data show that biomarker-selected pharmacology can improve body weight, appetite, and physical activity in a defined cachexia population, reinforcing the feasibility of mechanism-guided trials; however, this approach does not directly address ferroptotic host-tissue injury or the tumor-host redox interface.^7^ Thus, CC should be viewed not as starvation in the presence of cancer but as a tumor-host disorder in which inflammatory cytokines, hepatic and adipose metabolic rewiring, neuroendocrine stress, mitochondrial dysfunction, and persistent skeletal-muscle catabolism reinforce one another.^8^^,^^9^^,^^10^

Ferroptosis is a useful framework for revisiting CC because it connects five processes already central to the syndrome: iron redistribution, phospholipid peroxidation, mitochondrial stress, inflammatory amplification, and loss of antioxidant buffering. Importantly, each process is pharmacologically accessible. Labile iron can be redistributed or chelated, lipid radical propagation can be interrupted, cystine-GSH-GPX4 capacity can be supported, and GPX4-independent defense systems can be exploited or spared. Ferroptosis is defined as iron-dependent regulated cell death driven by lethal phospholipid peroxidation.^11^^,^^12^^,^^13^ Its major defense modules are distinct druggable nodes: the cystine-GSH-GPX4 axis detoxifies phospholipid hydroperoxides,^14^^,^^15^^,^^16^ whereas FSP1-CoQ10,^17^^,^^18^ DHODH-CoQH2,^19^ GCH1-BH4,^20^ and 7-DHC/DHCR7-dependent sterol defenses^21^^,^^22^ provide parallel or compartment-specific buffering. These pathways have been studied most intensively in tumor biology, where ferroptosis induction may overcome therapy resistance and interact with antitumor immunity.^23^^,^^24^^,^^25^ The same biochemical requirements, redox-active iron, PUFA-rich membranes, and limited antioxidant reserve, are also present in cachexia-vulnerable host tissues, especially skeletal muscle and adipose tissue.

The strongest cachexia-specific evidence currently comes from three mechanistic observations. First, in lung CC, tissue-infiltrating neutrophils release lipocalin-2 (LCN2), which drives iron-dependent lipid peroxidation and ferroptotic injury in adipose tissue and skeletal muscle; LCN2 blockade, neutrophil depletion, iron chelation, or ferroptosis inhibition mitigates wasting and improves survival in preclinical models.^26^ Second, in pancreatic cancer-associated cachexia, tumor-derived exosomal miR-203a-3p induces skeletal muscle ferroptosis through a ZEB1-SLC11A2/DMT1 iron-import program, and ferroptosis blockade attenuates muscle loss despite ongoing tumor burden.^27^ Third, ketogenic diet combined with GPX4 inhibition enhances tumor ferroptosis but can precipitate endocrine stress, weight loss, and muscle atrophy, shortening survival unless systemic adaptation is restored.^23^

These emerging studies reposition ferroptosis from a tumor-intrinsic death mechanism to a tumor-host pharmacology interface. The field, nevertheless, faces an interpretive problem. Many cachexia studies have inferred ferroptosis from elevated iron, malondialdehyde (MDA), 4-hydroxynonenal, lipid reactive oxygen species (ROS), and ACSL4 expression or reduced GPX4 expression. These readouts are informative but not individually specific; they may also reflect secondary oxidative injury, malnutrition, mitochondrial stress, treatment toxicity, or inflammation. A rigorous review should, therefore, distinguish druggable causal mechanisms from associative signatures and avoid using ferroptosis as a convenient umbrella term for all cachexia-related oxidative damage. Throughout this review, we distinguish direct evidence of ferroptotic execution from indirect ferroptosis-associated alterations. Direct evidence requires functional validation of ferroptotic mechanisms, whereas commonly reported changes such as iron accumulation, lipid peroxidation, or altered ferroptosis-related proteins should be interpreted cautiously unless supported by additional mechanistic validation. We first summarize ferroptosis-related mechanisms potentially relevant to wasting tissues, then organize current evidence according to tissue compartments and causal strength. Finally, we propose a hypothesis-generating split-ferroptosis framework: tumor ferroptosis sensitivity may be therapeutically exploited, while ferroptosis-related vulnerability in host metabolic tissues may require concurrent protection.

## Ferroptosis machinery most relevant to cachexia

### Peroxidizable membrane lipids: The substrate problem

Ferroptosis execution depends on the availability and oxidation of PUFA-containing phospholipids, particularly phosphatidylethanolamines (PEs) enriched in arachidonic or adrenic acid. ACSL4 activates free PUFAs to PUFA-CoA, and LPCAT3 incorporates them into membrane phospholipids, generating substrates that can be oxidized enzymatically by lipoxygenases^28^^,^^29^ or non-enzymatically through iron-catalyzed radical chemistry.^14^^,^^30^ Once phospholipid hydroperoxides exceed the repair capacity, membrane integrity fails, and regulated cell death follows. In cachexia, this substrate problem is not restricted to malignant cells. Muscle and adipose tissue undergo remodeling of lipid uptake, storage, mobilization, and oxidation. Enhanced adipose lipolysis and browning increase the fatty-acid flux,^31^^,^^32^^,^^33^ whereas altered muscle lipid remodeling and mitochondrial ROS can increase peroxidizable phospholipid pressure^34^^,^^35^ (Figure 1A). These changes do not prove ferroptosis, but they create a permissive lipid environment in which inflammatory or tumor-derived iron signals may lower the threshold for ferroptosis-like injury.

**Figure 1:**
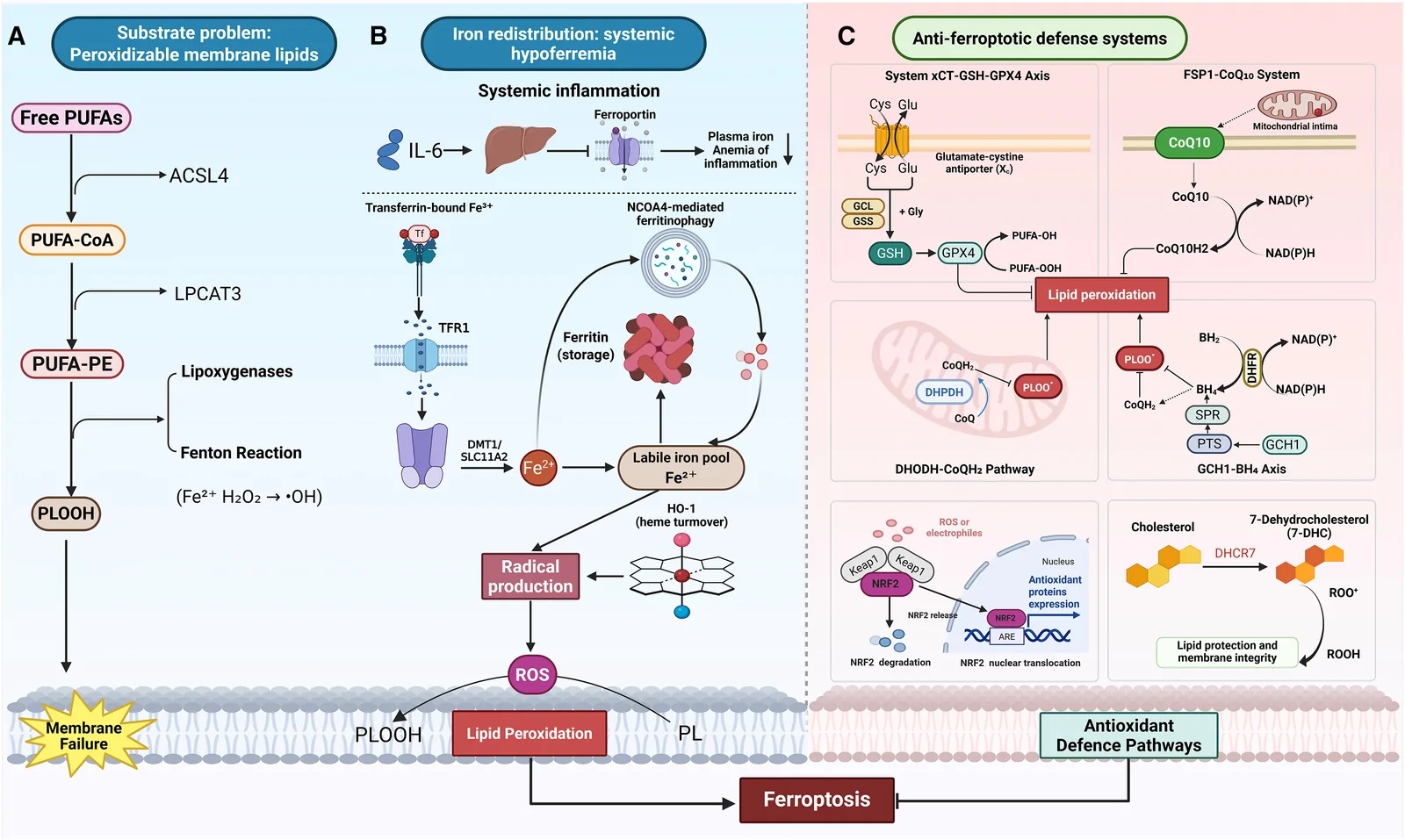
Core ferroptosis machinery most relevant to cancer cachexia (A) Peroxidizable membrane lipid substrates arise from PUFA activation by ACSL4 and incorporation by LPCAT3 into PUFA-containing phospholipids, which can be oxidized by lipoxygenases or iron-dependent Fenton chemistry to form phospholipid hydroperoxides and membrane damage. (B) Inflammatory iron redistribution involves IL-6/hepcidin/ferroportin signaling, transferrin receptor- and DMT1/SLC11A2-mediated iron import, ferritin storage/ferritinophagy, and heme turnover, thereby expanding local labile iron and radical production. (C) Anti-ferroptotic defense systems, including the system x_cˆ-/GSH/GPX4, FSP1-CoQ10, DHODH-CoQH2, GCH1-BH4, NRF2, and 7-DHC/DHCR7 pathways, buffer lipid peroxidation and maintain membrane integrity.

### Iron redistribution: Systemic hypoferremia can coexist with tissue iron stress

Iron is required for ferroptosis because redox-active Fe^2+^ supports Fenton chemistry and lipid radical propagation. Cellular iron availability is governed by transferrin receptor 1 (TFR1), DMT1/SLC11A2, ferritin, ferroportin, and heme turnover through heme oxygenase-1.^36^^,^^37^^,^^38^ NCOA4-mediated ferritinophagy is a particularly relevant route by which ferritin-bound iron enters the labile iron pool (LIP).^39^ Inflammation imposes systemic regulation through the IL-6-hepcidin-ferroportin axis, which promotes sequestration of iron in macrophages and hepatocytes and contributes to anemia of inflammation^40^^,^^41^^,^^42^ (Figure 1B). A central cachexia nuance is that inflammatory hypoferremia and tissue iron stress can coexist. Skeletal muscle and adipose tissue may expand their local LIPs, even when circulating iron is low. LCN2 is especially relevant because it redistributes iron and directly mediates ferroptotic wasting in lung CC.^26^ Similarly, increased DMT1/SLC11A2 expression in pancreatic cancer-associated muscle wasting supports the view that local iron import, not systemic iron overload alone, determines ferroptotic susceptibility.^27^ This tension argues against indiscriminate iron manipulation in cachectic patients.

### Anti-ferroptotic defense systems: GPX4-dependent and GPX4-independent buffering

The system Xc⁻–GSH-GPX4 axis remains the best characterized anti-ferroptotic pathway. SLC7A11/SLC3A2 imports cystine, cystine supports glutathione (GSH) biosynthesis, and GPX4 reduces phospholipid hydroperoxides to non-toxic alcohols.^14^^,^^15^^,^^16^ Nutrient deprivation, inflammatory stress, oxidative burden, and impaired amino acid availability can weaken this axis, rendering metabolically stressed tissues more sensitive to lipid peroxidation. GPX4-independent systems provide additional protection. FSP1 regenerates ubiquinol at the plasma membrane and suppresses lipid radical propagation, even when GPX4 is compromised.^17^^,^^18^ DHODH sustains reduced CoQ pools in the inner mitochondrial membrane and can compensate for low mitochondrial GPX4 activity.^19^^,^^43^ GCH1-derived BH4 acts as a radical-trapping antioxidant (RTA) and shapes lipid remodeling through interactions with the ubiquinone pool.^20^ Emerging studies suggest that sterol metabolism may influence ferroptosis sensitivity, with the 7-DHC-DHCR7 axis representing a potential but still evolving regulatory pathway.^21^^,^^22^ NRF2 integrates redox defense, GSH metabolism, ferritin storage, and heme turnover, yet its therapeutic activation is double-edged because it can enhance both host stress tolerance and tumor cell survival.^44^^,^^45^ These core substrate, iron-handling, and defense modules define the druggable ferroptosis nodes summarized in Figure 1C. For cachexia, the key question is not whether these pathways exist, but whether they fail selectively in skeletal muscle and adipocytes. Lipid-peroxide stress may impair contractility, mitochondrial performance, adipocyte function, or regeneration before overt cell death is visible. The ferroptosis-cachexia axis is, therefore, best understood as a continuum from sublethal phospholipid damage to regulated cell death.

## Evidence for ferroptosis in cachexia-affected tissues

### Interpreting the evidence: From signatures to causality

Ferroptosis is often inferred from a marker cluster: increased Fe^2+^ or labile iron, elevated MDA, 4-HNE, lipid ROS, or oxidized PE species, increased ACSL4/ALOX15/TFR1/DMT1, decreased SLC7A11 or GPX4, and rescue by ferroptosis inhibitors or iron chelators. These readouts are useful, but only intervention-based rescue and genetic or cell-type-specific perturbation provide stronger causal evidence.^13^^,^^46^^,^^47^ In CC, this distinction matters because inflammation, starvation, mitochondrial dysfunction, and treatment toxicity can all produce lipid damage without canonical ferroptotic cell death.^48^^,^^49^^,^^50^ Table 1, therefore, ranks the current evidence by causal strength rather than by marker abundance. At present, the most direct cachexia-specific evidence involves immune-derived LCN2 and tumor-derived exosomal miRNA signaling. Evidence from general muscle atrophy, adipose remodeling, and CNS anorexia models is biologically informative but should not be over-extrapolated to CC without rescue experiments and tissue-specific validation.

**Table 1:** Evidence hierarchy for ferroptosis involvement in cancer cachexia

Model or context	Affected tissue	Ferroptosis-related evidence	Intervention or validation	Interpretation	References
Lung cancer cachexia	adipose tissue and skeletal muscle	neutrophil infiltration, LCN2 elevation, iron accumulation, lipid peroxidation, and reduced GPX4 expression	myeloid *Lcn2* deletion, LCN2 neutralization, neutrophil depletion, liproxstatin-1, and deferoxamine	direct evidence that immune-derived LCN2 promotes ferroptotic wasting	Wang et al.^26^
Pancreatic cancer-associated cachexia	skeletal muscle	tumor exosomal miR-203a-3p, ZEB1-SLC11A2/DMT1 iron-import axis, lipid peroxidation, and GPX4 suppression	ferroptosis blockade attenuates muscle wasting	strong tumor-host mechanism, currently cancer type specific	Hu et al.^27^
Sarcopenia, disuse, sepsis, chemotherapy exposure, and other catabolic states	skeletal muscle	iron accumulation, lipid hydroperoxides, altered ACSL4/SLC7A11/GPX4, and mitochondrial stress	ferroptosis inhibitors, iron chelation, or antioxidant interventions partially preserve mass/function	supports biological plausibility but requires cachexia-specific validation	Weber et al.,^33^ Sun et al.,^34^ Beltrà et al.,^51^ Wang et al.,^52^ Huang et al.,^53^ Eshima et al.,^54^ Czyżowska et al.,^55^ and Thakir et al.^56^
Lung cancer cachexia and adipose remodeling models	white adipose tissue	Fe^2+^ accumulation, MDA/lipid peroxidation, LCN2 signaling, and immune infiltration	LCN2 blockade, iron chelation, and ferroptosis inhibition	ferroptosis likely interacts with lipolysis and browning, rather than replacing them	Hassannia et al.,^25^ Conrad, and Pratt^30^ Kir et al.,^31^ Das et al.,^32^ Yang et al.,^57^ Babic et al.,^58^ and Lefevre et al.^59^
Ketogenic diet plus GPX4 inhibition	whole organism, tumor and host endocrine-metabolic tissues	enhanced tumor ferroptosis with systemic weight loss, muscle atrophy, and endocrine stress	dexamethasone partially restores systemic adaptation without abolishing tumor response	demonstrates tumor-host ferroptosis trade-off	Li et al.^22^

### Skeletal muscle wasting: Ferroptosis as an amplifier of catabolic injury

Skeletal muscle loss is the defining clinical feature of CC. Established mechanisms include ubiquitin-proteasome activation, autophagy-lysosome signaling, impaired protein synthesis, mitochondrial dysfunction, and defective regeneration.^51^^,^^60^^,^^61^ Ferroptosis does not replace these mechanisms; it may amplify them by converting inflammatory iron stress and phospholipid peroxidation into persistent myofiber injury.^34^^,^^52^ Across non-cancer catabolic models, atrophic muscle frequently shows iron accumulation, lipid peroxide formation, GPX4 impairment, and partial rescue by ferroptosis inhibitors or iron chelation.^53^^,^^54^^,^^55^ These findings show that skeletal muscle is competent to undergo ferroptosis-relevant injury under metabolic stress, but cachexia-specific proof remains essential. In CC, two mechanisms stand out. In lung CC, infiltrating neutrophils secrete LCN2, increasing labile iron and lipid peroxidation in skeletal muscle; genetic or pharmacological interruption of this axis attenuates wasting.^26^ In pancreatic cancer-associated cachexia, tumor-derived exosomal miR-203a-3p promotes myofiber ferroptosis by inducing DMT1/SLC11A2-dependent iron uptake through a ZEB1-linked mechanism.^27^ Together, these studies support the concept that tumor and immune signals can converge on iron handling within myofibers (Figure 2A).

**Figure 2:**
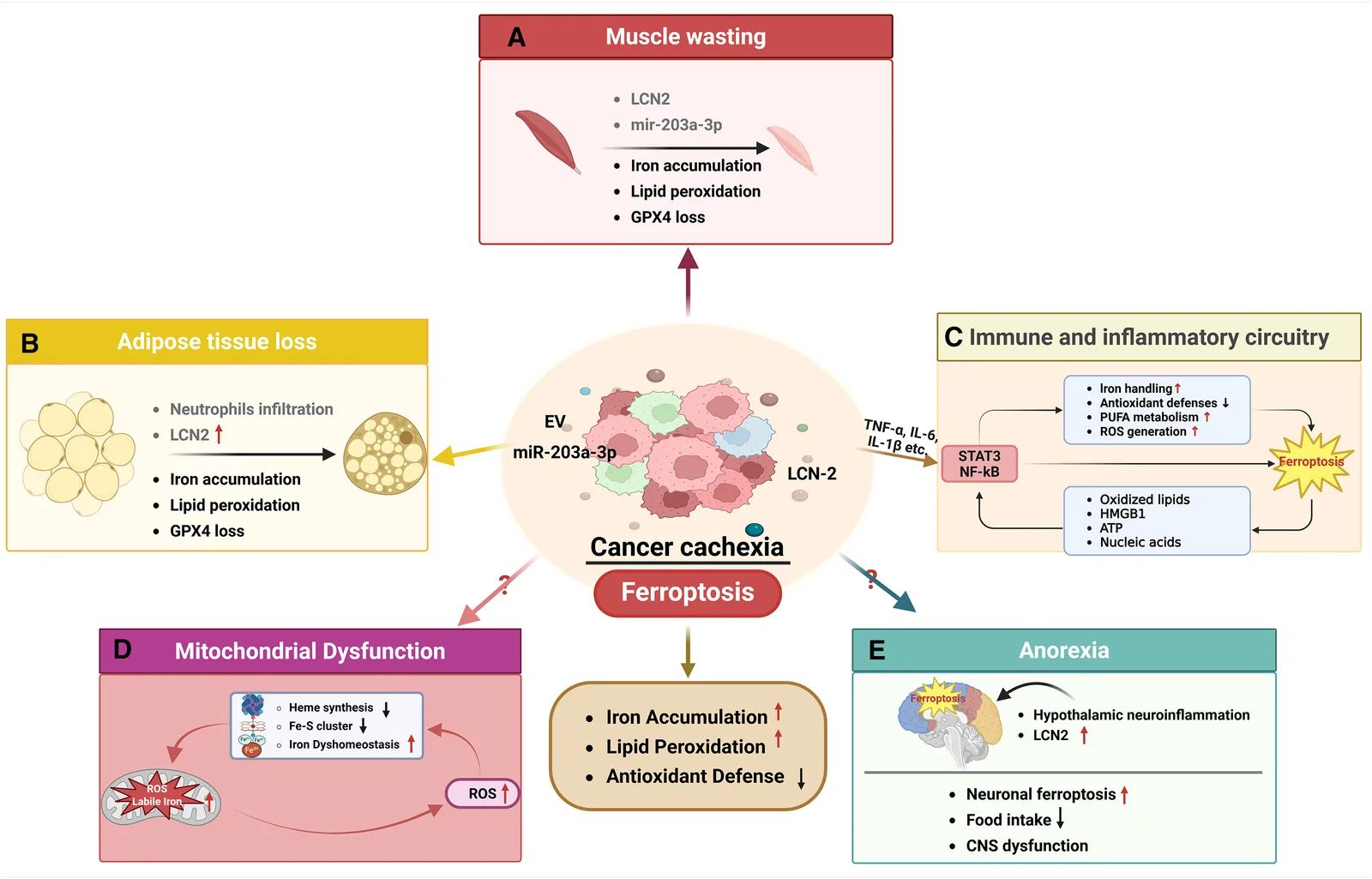
Cachexia-specific ferroptosis mechanisms across host tissues (A) Skeletal muscle wasting is linked to LCN2- and tumor-derived miR-203a-3p-associated iron accumulation, lipid peroxidation, and GPX4 loss. (B) Adipose tissue loss integrates neutrophil infiltration, LCN2 signaling, iron accumulation, lipid peroxidation, and impaired antioxidant defense. (C) Immune and inflammatory circuits connect TNF-α, IL-6, IL-1β, STAT3/NF-κB activation, iron handling, PUFA metabolism, and oxidized lipid/DAMP release. (D) Mitochondrial dysfunction connects labile iron, ROS production, heme/Fe-S cluster disturbance, and iron dyshomeostasis. (E) Anorexia is presented as a putative central component involving hypothalamic neuroinflammation, LCN2, neuronal lipid peroxidation stress, reduced food intake, and CNS dysfunction.

Ferroptosis also intersects with canonical muscle catabolism. Iron-driven oxidative stress can activate NF-κB, p38 MAPK, and related inflammatory pathways that induce atrogin-1 and MuRF1, linking lipid peroxidation to proteolytic machinery.^34^^,^^56^ NCOA4-mediated ferritinophagy expands the LIP and may accelerate ferroptotic vulnerability,^39^^,^^62^ whereas dysregulated autophagy and mitochondrial turnover can maintain ROS-leaking organelles.^51^^,^^57^^,^^63^ Lipid hydroperoxides and carbonyl stress impair muscle homeostasis and promote weakness, suggesting that ferroptosis-associated lipid damage may degrade muscle function before irreversible cell death is evident.^35^^,^^54^ A crucial unresolved issue is whether ferroptosis primarily kills myofibers or creates a sublethal lipid peroxidation state that impairs contraction, mitochondrial energetics, and repair. Future studies should, therefore, measure contractile function, mitochondrial respiration, regeneration markers, and ferroptosis-rescue effects together, rather than relying on lipid peroxidation markers alone.

### Adipose tissue loss: Regulated cell death layered onto lipolysis and browning

Adipose tissue depletion often precedes clinically apparent muscle wasting and contributes to systemic energy failure.^58^ Traditional explanations emphasize adipose triglyceride lipase (ATGL)/hormone-sensitive lipase (HSL)-dependent lipolysis, impaired adipogenesis, WAT browning, thermogenesis, and inflammatory remodeling.^31^^,^^32^^,^^33^ Ferroptosis adds a complementary layer: adipocytes contain abundant oxidizable lipids and are exposed to inflammatory and immune-derived iron signals, making them vulnerable when antioxidant defenses are insufficient. The most direct evidence again comes from lung CC, in which adipose tissue exhibits Fe^2+^ accumulation, lipid peroxidation, ferroptosis-associated transcriptional changes, and protection by ferroptosis inhibition or iron chelation^26^ (Figure 2B). Neutrophil-derived LCN2 is mechanistically important because it reshapes local iron availability and promotes both adipose and muscle wasting. Thus, adipose loss in CC is not simply triglyceride mobilization; it may also involve regulated oxidative cell injury.

Ferroptosis is likely to cooperate with, rather than substituting for, canonical adipose wasting programs. Enhanced lipolysis increases fatty acid availability, WAT browning raises mitochondrial ROS, and inflammatory cytokines weaken redox buffering.^56^^,^^59^^,^^64^ These events can increase peroxidizable lipid substrates and accelerate lipid radical propagation. Conversely, ferroptotic adipocyte injury may release oxidized lipid mediators and inflammatory signals that further stimulate local macrophages, systemic inflammation, and muscle catabolism. This bidirectional model now needs direct testing using adipocyte-specific genetic models, depot-resolved lipidomics, and functional measures of whole-body energy balance.

### Immune and inflammatory circuitry: The ferroptosis-inflammation loop

Chronic inflammation is a defining feature of CC, and ferroptosis is both regulated by and capable of amplifying inflammatory signaling. IL-6, TNF-α, JAK-STAT3, and NF-κB pathways can alter iron handling, antioxidant defenses, PUFA metabolism, and ROS generation.^65^^,^^66^^,^^67^ The same pathways drive muscle proteolysis, hepatic acute-phase responses, adipose remodeling, anorexia, and metabolic dysfunction in cachexia.^9^^,^^60^^,^^61^ Their convergence with ferroptosis provides a plausible mechanism by which inflammatory tone lowers the threshold for tissue lipid peroxidation. Ferroptotic cells can release DAMPs and oxidized lipids, HMGB1, ATP, and nucleic acids that activate innate immune receptors and inflammasome-related pathways^46^^,^^68^^,^^69^ (Figure 2C). In this context, ferroptosis is not merely an endpoint; it can become an inflammatory amplifier. The LCN2-neutrophil axis is the clearest CC example, showing how immune infiltration transmits tumor-associated stress to peripheral metabolic tissues and drives iron-dependent injury.^26^ Whether macrophages, myeloid-derived suppressor cells, or therapy-induced immune remodeling produce similar loops remains unresolved.

### Mitochondrial dysfunction: Convergence without oversimplification

Mitochondrial dysfunction is one of the most reproducible features of cachectic muscle. Reduced biogenesis, impaired oxidative phosphorylation, excessive fission, defective mitophagy, and persistent mitochondrial ROS contribute to fatigue, weakness, and proteolytic activation.^51^^,^^63^ Ferroptosis intersects with this network because mitochondrial iron handling, Fe-S cluster synthesis, heme metabolism, and lipid peroxidation all influence the membrane redox state^57^^,^^70^^,^^71^ (Figure 2D). However, ferroptosis should not be used as a synonym for mitochondrial oxidative stress. Some ferroptosis programs require mitochondria, whereas others are driven mainly by plasma membrane lipid oxidation or extramitochondrial iron chemistry. In cachexia, mitochondrial ROS may sensitize tissues to ferroptosis by promoting lipid peroxidation, while ferroptosis-associated lipid damage may further impair respiratory complexes and membrane integrity. This reciprocal model is more defensible than a simple claim that mitochondrial dysfunction equals ferroptosis.

### Anorexia and central regulation: A hypothesis requiring direct evidence

Anorexia is an early and clinically important component of CC. It involves inflammatory cytokines, hypothalamic and brainstem appetite circuits, melanocortin signaling, vagal inputs, treatment toxicity, and peripheral endocrine mediators such as GDF15 and LCN2.^72^^,^^73^^,^^74^ Hypothalamic and brainstem neuroimmune remodeling provides an additional link between tumor-derived signals and appetite control.^73^^,^^75^^,^^76^ Neurons and glia are rich in PUFAs and rely on tight iron and redox regulation, making ferroptosis-relevant lipid peroxidation biologically plausible in central feeding networks.^77^^,^^78^^,^^79^ Direct evidence that overt hypothalamic ferroptotic cell death causes cancer-associated anorexia is, nevertheless, limited. A more cautious interpretation is that ferroptosis-relevant oxidative lipid stress may modulate neuroimmune signaling, synaptic function, or glial activation in appetite-regulatory regions. This hypothesis should be tested using brain-region-specific lipidomics, iron imaging, ferroptosis-rescue experiments, and behavioral endpoints. Until then, CNS ferroptosis should be presented as a potential modifier, rather than an established driver of cachexia-associated anorexia (Figure 2E).

## Tumor ferroptosis and CC: Pharmacological therapeutic index and paradox

Tumor biology often frames ferroptosis induction as desirable because it can kill therapy-resistant cancer cells and cooperate with antitumor immunity.^24^^,^^25^ CC requires a more stringent pharmacological interpretation. Cachexia-affected host tissues share several ferroptosis vulnerabilities with tumors: PUFA-rich membranes, inflammatory oxidative stress, iron redistribution, and limited antioxidant reserve. Systemic ferroptosis induction may, therefore, improve tumor control, while worsening muscle and adipose loss. The ketogenic diet plus GPX4 inhibition study illustrates this problem: the intervention promoted tumor ferroptosis but induced rapid weight loss, muscle atrophy, and endocrine disruption and reduced survival; dexamethasone partially restored systemic adaptation without fully abolishing the antitumor ferroptotic efficacy.^23^ This study makes an important point for drug development: ferroptosis-sensitizing strategies act on the organism, not only on malignant cells. Endocrine, nutritional, and metabolic support may, therefore, be necessary when ferroptosis-based oncology interventions are applied to patients already at risk of cachexia.

Pharmacological inhibition of xCT/SLC7A11 or GPX4 raises similar concerns. These targets are attractive in cancer cells but are also host antioxidant defenses. Skeletal muscle and adipose tissue may become collateral targets, particularly in patients with inflammation, malnutrition, low antioxidant reserve, or pre-existing weight loss.^52^^,^^56^^,^^80^^,^^81^ Conversely, broad ferroptosis inhibition could protect host tissues but blunt antitumor immunity or reduce therapy sensitivity. The therapeutic goal is, therefore, neither global induction nor global inhibition; it is spatial, temporal, and pharmacological separation of tumor and host ferroptosis responses. We refer to this as a split-ferroptosis paradigm. In tumors, ferroptosis may be intentionally induced or sensitized when it improves oncologic response. In host metabolic tissues, ferroptosis resistance should be preserved through iron modulation, lipid peroxide scavenging, nutritional support, and reinforcement of antioxidant defense. Achieving this separation will require tissue-selective delivery, careful scheduling, patient stratification, and biomarker-guided monitoring of tumor response and host tissue integrity.

## Pharmacological strategies: From anti-ferroptotic rescue to tumor-host split-ferroptosis control

### Iron modulation and the LCN2-DMT1 axis

Iron chelation is a direct way to reduce labile iron availability and suppress iron-catalyzed lipid peroxidation. In preclinical lung CC, deferoxamine (DFO) attenuated ferroptosis-associated markers and tissue wasting.^26^ For translational pharmacology, however, iron modulation should be framed as target-context pharmacology rather than generic chelation. Clinically available chelators may protect host tissues, but they can worsen anemia, alter infection risk, interact with renal or hepatic dysfunction, and interfere with tumor-directed therapy. Cachexia may involve tissue-specific iron redistribution, rather than systemic iron excess. LCN2 and DMT1/SLC11A2, therefore, represent more disease-proximal targets. LCN2 connects neutrophil infiltration to tissue iron dysregulation in lung CC,^26^ whereas DMT1/SLC11A2 links tumor-derived exosomal miR-203a-3p to pancreatic cancer-associated muscle ferroptosis^27^ (Figure 3A). Selective modulation of these pathways could preserve host tissues, while avoiding indiscriminate systemic iron depletion. However, both molecules participate in immune defense, iron handling, and tumor biology; so, tissue context, timing, and pharmacodynamic monitoring will be critical.

**Figure 3:**
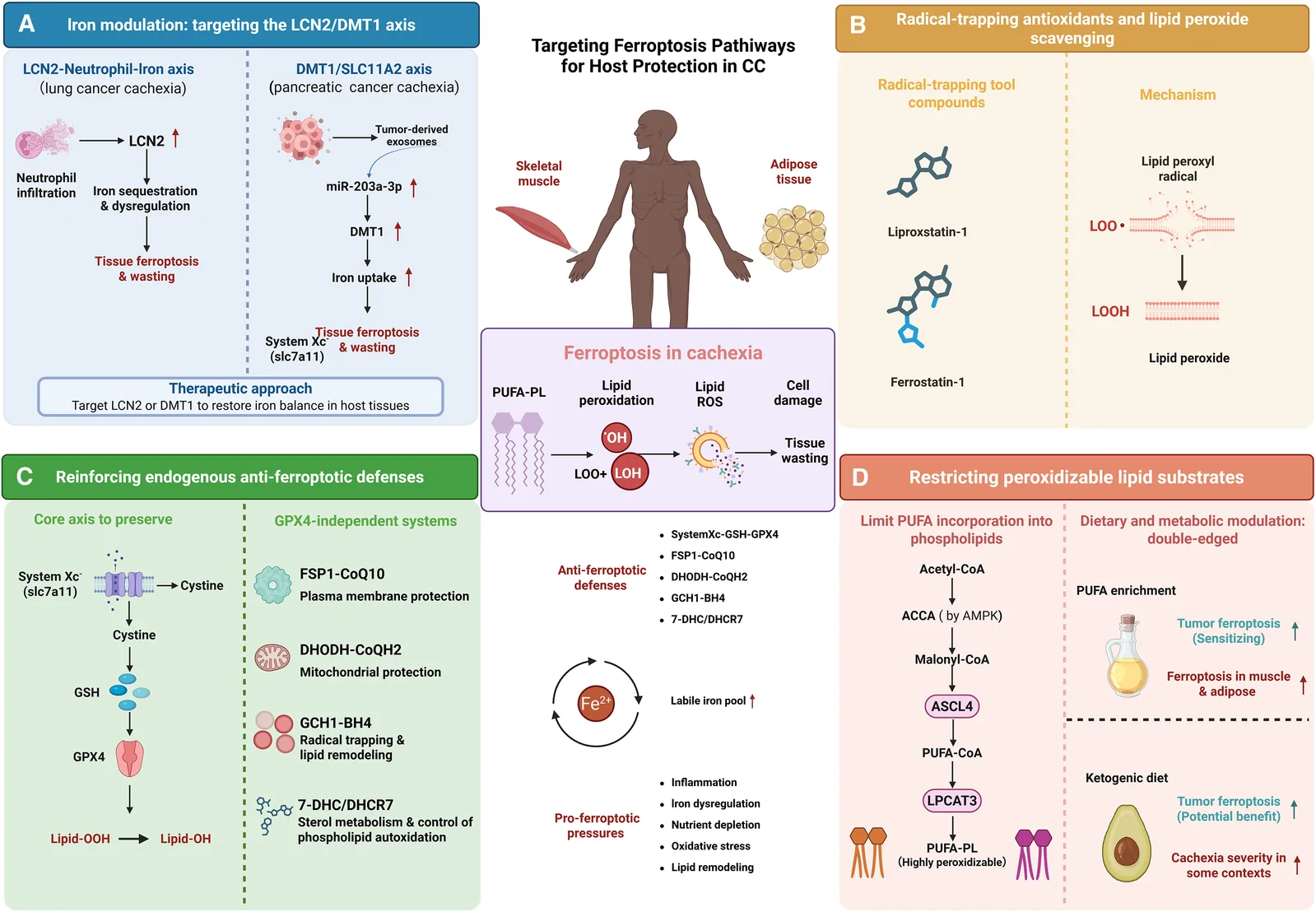
Pharmacological target map for split-ferroptosis control The map separates tumor-directed sensitization, including xCT inhibition, GPX4 inhibition, increased labile iron, PUFA sensitization and therapy/immunotherapy synergy, from host-protective strategies, including LCN2 blockade, DMT1 restriction, selective iron modulation, radical-trapping antioxidants, CoQ10/BH4/mitochondrial redox support and nutritional redox support. The central modules-iron handling, PUFA-phospholipid remodeling, xCT/SLC7A11, GPX4-GSH, GPX4-independent defenses and LCN2-mediated iron stress - illustrate how the same ferroptosis machinery can be exploited to enhance tumor ferroptosis while preserving skeletal muscle and adipose tissue function.

### RTAs and lipid peroxide scavenging

Liproxstatin-1 and ferrostatin-1 are widely used tool compounds that suppress lipid radical propagation and remain central to ferroptosis validation.^82^^,^^83^ Liproxstatin-1 protects wasting tissues in lung CC models^26^ (Figure 3B). Although these compounds are not established cachexia therapeutics, they provide proof of principle that lipid peroxide scavenging can mitigate tissue wasting under ferroptosis-permissive conditions. Translation will require drug-like RTAs with adequate exposure in skeletal muscle and adipose tissue, acceptable safety during cancer therapy, and limited tumor-protective activity. Clinically accessible lipophilic antioxidants such as vitamin E may overlap mechanistically with radical trapping,^83^ but their potency, tissue distribution, and selectivity remain uncertain. Antioxidants used without biomarker guidance are unlikely to succeed because cachexia has multiple parallel drivers. A rational approach would combine lipid peroxide monitoring with defined intervention windows, functional endpoints, and simultaneous assessment of tumor response.

### Reinforcing endogenous anti-ferroptotic defenses

Preserving cysteine availability, GSH synthesis, and GPX4 activity is a plausible host-protective strategy. Nutritional depletion and inflammation may weaken the system x_cˆ--GSH-GPX4 axis, and cysteine precursors or redox-supportive nutrients could theoretically raise ferroptosis resistance in vulnerable tissues.^84^ Yet, systemic reinforcement of antioxidant defenses can also protect tumors. This problem is especially relevant for NRF2, which may enhance host redox resilience and tumor stress tolerance at the same time.^44^^,^^45^ GPX4-independent systems offer additional pharmacological entry points: FSP1-CoQ10 protects plasma membranes,^17^^,^^18^ DHODH-CoQH2 supports mitochondrial defense,^19^^,^^43^ GCH1-BH4 contributes radical-trapping and lipid-remodeling capacity,^20^ and the 7-DHC-DHCR7 axis has been proposed as a sterol-metabolic regulator of ferroptosis sensitivity, although its broader biological and translational relevance requires further validation^21^^,^^22^ (Figure 3C). CoQ-related strategies, mitochondrial redox support, BH4-linked modulation, and 7-DHC/DHCR7 manipulation are attractive but double edged. Cachexia-specific studies should determine whether these defenses fail in skeletal muscle and adipose tissue, and whether selective activation preserves host function without accelerating tumor growth or therapy resistance.

### Restricting peroxidizable lipid substrates

Because ferroptosis sensitivity depends on membrane PUFA-phospholipid content, reducing peroxidizable substrates is conceptually attractive. ACSL4 and LPCAT3 regulate PUFA incorporation into phospholipids and shape ferroptosis susceptibility.^28^^,^^29^^,^^85^ The AMPK-ACC-ACSL4 axis has been implicated in muscle atrophy and may provide a metabolic entry point for lowering PUFA-phospholipid peroxidizability.^86^ Dietary manipulation requires particular caution. PUFA enrichment can sensitize tumors to ferroptosis,^87^^,^^88^ but systemic PUFA availability may also reduce ferroptosis thresholds in muscle and adipose tissue. Likewise, ketogenic diets can alter lipid flux and redox balance in ways that amplify tumor ferroptosis while aggravating cachexia in specific therapeutic contexts^23^ (Figure 3D). Nutritional interventions should, therefore, be evaluated as pharmacometabolic modulators, not as benign background care, and should be paired with body composition, muscle function, endocrine status, lipid peroxidation biomarkers, and tumor response.

### Scheduling, delivery, and combination therapy

The potential implementation of a split-ferroptosis strategy may require approaches that achieve spatial or temporal separation of ferroptotic modulation between tumor and host tissues. Three principles are especially relevant. First, tumor-directed ferroptosis inducers should be scheduled away from periods of maximal host vulnerability, such as rapid weight loss, systemic inflammation, endocrine stress, chemotherapy-related anorexia, or postoperative catabolism. Second, delivery systems should enrich ferroptosis sensitizers in tumors while sparing skeletal muscle, adipose tissue, and neuroendocrine circuits. Third, host-protective interventions should be developed as components of combination regimens, rather than as isolated supportive care. This requires co-reporting of tumor response, lean mass, adipose mass, muscle strength, appetite, inflammatory burden, treatment completion, and survival. Current cachexia guidelines, multimodal studies, and biomarker-selected pharmacological trials support the need for clinically meaningful endpoints that go beyond body weight alone.^4^^,^^5^^,^^6^^,^^89^^,^^90^^,^^91^ The principal target classes and safety liabilities are summarized in Figure 4 and Table 2. Immune-mediated ferroptosis represents another important layer of tumor-host interaction. CD8^+^ T cell-derived interferon γ (IFN-γ) has been reported to enhance tumor ferroptosis by suppressing ferroptosis-protective pathways, providing a mechanistic link between antitumor immunity and ferroptotic vulnerability. However, whether immune-driven ferroptosis during immunotherapy influences systemic wasting or alters ferroptosis susceptibility in host metabolic tissues remains largely unexplored. Future studies should determine whether immunotherapy-associated ferroptosis can be integrated into a tumor-selective ferroptosis framework, while preserving host tissue integrity.

**Figure 4:**
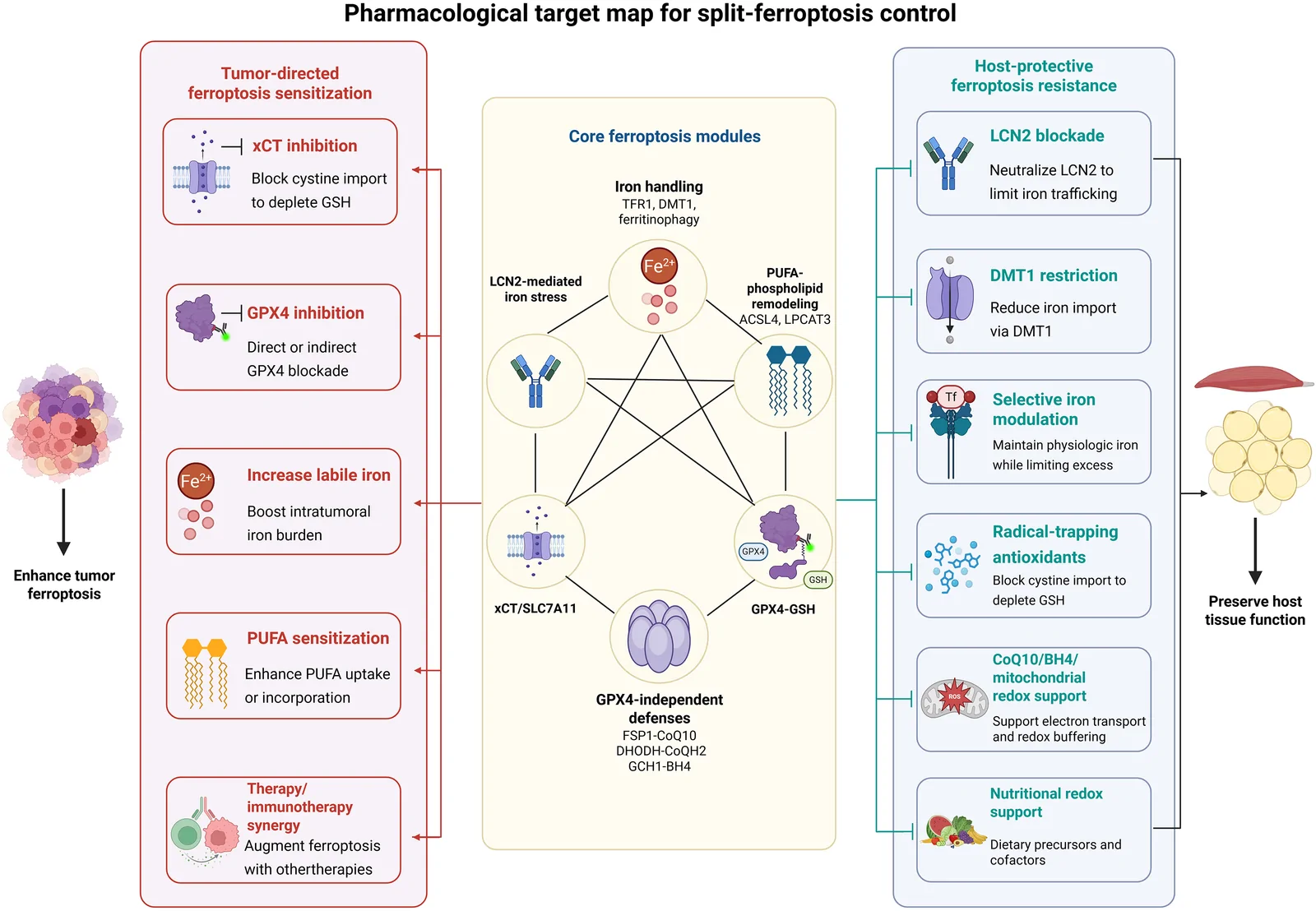
Host-protective pharmacological interventions targeting ferroptosis pathways in cancer cachexia (A) Iron modulation centers on the neutrophil-LCN2-iron axis in lung cancer cachexia and the tumor-derived exosomal miR-203a-3p-DMT1/SLC11A2 axis in pancreatic cancer-associated muscle wasting, highlighting LCN2 blockade and DMT1 restriction as disease-proximal strategies to normalize local iron stress. (B) Radical-trapping antioxidants such as liproxstatin-1 and ferrostatin-1 interrupt lipid peroxyl radical propagation and limit phospholipid hydroperoxide accumulation. (C) Reinforcement of endogenous anti-ferroptotic defenses includes preservation of the system x_cˆ--GSH-GPX4 axis and GPX4-independent FSP1-CoQ10, DHODH-CoQH2, GCH1-BH4, and 7-DHC/DHCR7 systems. (D) Restricting peroxidizable lipid substrates through ACSL4/LPCAT3-related PUFA-phospholipid remodeling or through carefully monitored dietary/metabolic modulation may protect skeletal muscle and adipose tissue but could also alter tumor ferroptosis sensitivity. The figure emphasizes that host protection should be biomarker guided and integrated with tumor-directed treatment, rather than being used as global ferroptosis suppression.

**Table 2:** Pharmacological opportunities and safety liabilities in split-ferroptosis control for cancer cachexia

Pharmacological node	Intended cachexia action	Key evidence or rationale	Main safety liability	References
LCN2-neutrophil axis	reduce tissue iron loading and lipid peroxidation in muscle/adipose depots	direct preclinical evidence in lung cancer cachexia with protection by LCN2 blockade, neutrophil depletion, liproxstatin-1, or DFO	may affect innate immunity, infection defense, and tumor-immune interactions	Wang et al.^26^
DMT1/SLC11A2 iron import	limit tumor-to-muscle iron transfer and ferroptotic myofiber injury	tumor exosomal miR-203a-3p induces muscle ferroptosis through ZEB1-SLC11A2/DMT1 signaling	systemic iron handling and anemia risk; cancer type specificity	Hu et al.^27^
Lipid peroxide scavengers/RTAs	interrupt phospholipid radical propagation in host tissues	liproxstatin-1 protects wasting tissues in lung cancer cachexia models	tumor protection if exposure is not host selective	Hassannia et al.,^25^ Liu et al.,^81^ and Scarpellini et al.^82^
GPX4, FSP1-CoQ10, DHODH-CoQH2, GCH1-BH4	reinforce endogenous anti-ferroptotic defense in metabolically stressed tissues	GPX4-dependent and -independent defenses buffer phospholipid hydroperoxides and lipid radicals	may enhance cancer cell stress tolerance or treatment resistance	Galluzzi et al.,^13^ Yang and Stockwell,^14^ Yang et al.,^15^ Friedmann Angeli et al.,^16^ Bersuker et al.,^17^ Doll et al.,^18^ Mao et al.,^19^ and Busti et al.^42^
7-DHC-DHCR7 sterol axis	modulate endogenous anti-autoxidation capacity	7-DHC suppresses ferroptosis by protecting phospholipids from autoxidation; pharmacological EBP/DHCR7 manipulation alters tumor and injury phenotypes	could promote ferroptosis resistance, tumor aggressiveness, or endocrine/sterol toxicity	Kraft et al.^20^ and Freitas et al.^21^
ACSL4/LPCAT3 and PUFA trafficking	lower peroxidizable phospholipid substrate availability in host tissues	ACSL4/LPCAT3 shapes ferroptosis-sensitive lipid composition	may blunt desired tumor ferroptosis or disrupt normal lipid metabolism	Hu et al.,^27^ Doll et al.,^28^ Ushmorov et al.,^84^ Jiang et al.,^85^ Liu et al.,^86^ and Mortensen et al.^87^
Timing plus multimodal support	expand tumor-host therapeutic index during ferroptosis-inducing oncology regimens	guidelines, multimodal cachexia studies, and the GDF15-directed ponsegromab trial support integrated pharmacological plus functional endpoints	complex trial design; endpoint heterogeneity	Roeland et al.,^4^^,^^5^ Arends et al.,^6^ Lupica-Tondo et al.,^88^ Temel et al.,^90^ and Bhatt et al.^99^

## Biomarkers and experimental standards for translation

A clinically useful ferroptosis-cachexia biomarker strategy must capture iron metabolism, lipid peroxidation, antioxidant defense, tissue wasting, and function. No single marker is sufficient. Ferritin, serum iron, transferrin saturation, hepcidin, CRP, IL-6, and LCN2 define the systemic iron-inflammation milieu.^41^^,^^42^^,^^88^^,^^92^ MDA and 4-HNE provide general lipid peroxidation readouts, whereas oxidized PE species are more specific to ferroptotic lipid chemistry when measured by targeted lipidomics.^93^^,^^94^ ACSL4, ALOX15, TFR1, DMT1/SLC11A2, SLC7A11, GPX4, FSP1, DHODH, GCH1, DHCR7, and NRF2-related targets can help characterize tissue susceptibility and defense capacity.^21^^,^^22^^,^^37^^,^^85^^,^^95^ For preclinical studies, the minimum validation standard should include (1) lipid peroxidation measurements beyond generic ROS; (2) iron or labile-iron assessment; (3) at least one anti-ferroptotic rescue intervention such as ferrostatin-1, liproxstatin-1, iron chelation, or genetic manipulation; (4) exclusion or parallel measurement of apoptosis, necroptosis, autophagy-dependent death, and nonspecific oxidative injury; and (5) functional outcomes such as grip strength, treadmill performance, *ex vivo* contractility, adipose depot mass, food intake, and survival.^12^^,^^13^ Electron microscopy and oxidized PE lipidomics can strengthen mechanistic attribution but should not replace causal rescue experiments. For clinical translation, biomarker panels should be paired with longitudinal body composition and performance measures. CT-derived muscle area and radiodensity, DEXA, muscle strength, physical performance, appetite scores, treatment tolerance, and survival should be integrated with blood and tissue biomarkers.^96^^,^^97^ Liquid biopsy approaches are attractive, particularly tumor-derived extracellular vesicle cargoes such as miR-203a-3p, which may identify patients whose muscle wasting is driven by tumor-to-muscle iron signaling.^27^ A proposed biomarker-guided workflow is shown in Table 3.

**Table 3:** Proposed biomarker and validation framework for ferroptosis in cancer cachexia

Domain	Representative markers or methods	What it indicates	Key limitation
Systemic iron inflammation	ferritin, serum iron, transferrin saturation, hepcidin, CRP, IL-6, and LCN2	inflammatory iron redistribution and ferroptosis-permissive context	does not prove tissue ferroptosis
Tissue iron stress	Fe^2+^ probes, LIP assays, TFR1, DMT1/SLC11A2, ferritin, ferroportin, and NCOA4	local iron availability and iron-handling imbalance	sampling and tissue heterogeneity
Lipid peroxidation	MDA, 4-HNE, lipid ROS probes, and oxidized PE-OOH lipidomics	core biochemical signal of ferroptosis	generic oxidative injury can overlap
Defense capacity	SLC7A11, GSH/GSSG, GPX4 activity, FSP1, CoQ10/CoQH2, DHODH, GCH1/BH4, and NRF2 targets	ability to buffer phospholipid hydroperoxides	compensatory changes may obscure directionality
Causal rescue	ferrostatin-1, liproxstatin-1, DFO, and genetic manipulation of LCN2, *DMT1, GPX4, ACSL4*, or *SLC7A11*	stronger evidence for ferroptosis contribution	tool compounds may have off-target effects
Clinical phenotype	CT-derived muscle area and radiodensity, adipose depot volume, DEXA, grip strength, performance tests, appetite, treatment tolerance, and survival	clinical relevance, prognostic value, and functional translation of ferroptosis-linked biology	requires longitudinal integration with molecular markers and body composition methodology

## Challenges and future directions

Several barriers must be resolved before ferroptosis can become a clinically actionable and pharmacologically useful pathway in CC. The first is mechanistic specificity. Iron accumulation, GPX4 reduction, ACSL4 induction, MDA formation, 4-HNE accumulation, or lipid ROS elevation should be treated as ferroptosis-associated evidence, rather than proof of ferroptotic cell death.^12^^,^^98^ Future studies should combine lipid peroxidation chemistry, labile iron assessment, ultrastructural analysis, rescue by ferroptosis inhibitors or iron chelation, and genetic perturbation of key nodes such as *GPX4*, *SLC7A11*, *ACSL4*, *LCN2*, *DMT1/SLC11A2*, *NCOA4*, or *DHCR7*.^12^^,^^18^^,^^21^^,^^22^^,^^26^^,^^27^^,^^28^^,^^49^^,^^98^ Investigators should also define whether ferroptosis initiates tissue wasting, amplifies established catabolism, or appears as a late consequence of inflammation, malnutrition, or anticancer therapy.^12^^,^^23^^,^^80^^,^^81^^,^^98^

A second priority is tissue, tumor, and patient heterogeneity. The available cachexia-specific evidence already indicates that ferroptosis is not uniform; lung CC can involve a neutrophil-LCN2 axis affecting adipose tissue and skeletal muscle,^26^ whereas pancreatic cancer-associated wasting can involve tumor-derived exosomal miR-203a-3p and DMT1/SLC11A2-dependent muscle iron import.^27^ Human skeletal muscle molecular subtyping further indicates that cachectic muscle is biologically diverse, rather than a single transcriptional state,^99^ and recent inter-organ analyses emphasize that liver, adipose tissue, muscle, immune cell, and tumor metabolism can be rewired in coordinated but model-dependent ways.^100^^,^^101^ Future work should stratify ferroptosis biology by the cancer type, disease stage, treatment exposure, sex, nutritional state, inflammatory status, and tissue compartment.

Third, the field needs temporal and spatial resolutions. Ferroptosis may be most reversible during a pre-cachectic or early inflammatory phase, when lipid peroxidation and iron stress are rising but irreversible myofiber loss, adipocyte depletion, and functional decline have not yet occurred.^12^^,^^26^^,^^27^^,^^98^ Longitudinal animal studies should pair body composition, food intake, contractile function, endocrine markers, immune profiling, and tissue-resolved lipidomics across defined time points.^100^^,^^101^ In parallel, clinical studies should test whether circulating LCN2, hepcidin, oxidized phospholipids, and extracellular vesicle cargoes such as miR-203a-3p identify patients with ferroptosis-permissive wasting biology.^26^^,^^27^^,^^100^

Fourth, therapeutic development must explicitly address the tumor-host paradox. Ferroptosis induction may be useful for tumor control and may interact favorably with chemotherapy, radiotherapy, targeted therapy, and antitumor immunity.^23^^,^^80^ However, the same ferroptotic pressure can compromise non-malignant tissues with high lipid flux or limited antioxidant reserve.^80^^,^^81^ The ketogenic diet plus GPX4 inhibition model illustrates that tumor ferroptosis can coincide with endocrine stress, rapid weight loss, muscle atrophy, and shortened survival unless host adaptation is supported.^23^ Adverse effects of ferroptosis-oriented therapies, including cachexia-related toxicity, are increasingly being recognized.^80^^,^^81^ Future studies should, therefore, report tumor response and host outcomes together, including lean mass, adipose mass, muscle strength, food intake, inflammatory burden, endocrine status, treatment tolerance, and survival.

Fifth, translational studies should move from single-marker association toward integrated decision frameworks. A useful early-phase trial would enroll patients at a high risk of cachexia, define ferroptosis-permissive biology using blood and tissue biomarkers, and test host-protective interventions without compromising antitumor efficacy.^26^^,^^27^^,^^80^^,^^81^^,^^100^ The ponsegromab trial illustrates the value of biomarker selection and patient-centered endpoints in cachexia pharmacology,^7^ while CT-based body composition studies show why lean mass and muscle quality should be measured, rather than inferred, from body weight or body mass index.^96^^,^^97^ Candidate ferroptosis-focused strategies include selective interruption of the LCN2 or DMT1/SLC11A2 axis, cautious iron modulation, lipid peroxide scavenging, preservation of cysteine-GSH-GPX4 capacity, activation of GPX4-independent defenses, and scheduling of tumor-directed ferroptosis therapies away from periods of maximal host vulnerability.^12^^,^^18^^,^^26^^,^^27^^,^^80^^,^^81^ These interventions should be judged by molecular markers, physical performance, treatment completion, patient-reported appetite and fatigue, hospitalization, and survival.

Finally, ferroptosis should be integrated into the broader cachexia network, rather than being treated as a stand-alone explanation. Proteolysis, impaired anabolism, mitochondrial dysfunction, autophagy, adipose lipolysis, WAT browning, hepatic metabolic reprogramming, anorexia, neuroinflammation, endocrine stress, microbiome-derived metabolites, and treatment toxicity all intersect with redox and iron metabolism.^100^^,^^101^^,^^102^ A promising hypothesis-generating direction is the exploration of a split-ferroptosis framework, in which tumor ferroptosis sensitivity may be enhanced when therapeutically beneficial, while vulnerable host tissues, including skeletal muscle, adipose tissue, immune compartments, and neuroendocrine circuits, are protected from ferroptosis-associated stress.^23^^,^^80^^,^^81^^,^^100^ Achieving this goal requires advances in tissue-selective delivery, longitudinal biomarker development, multi-omics validation, and combination strategies evaluated at the level of the whole organism rather than the tumor alone. This framework positions ferroptosis in CC as a translational challenge centered on the therapeutic index, patient stratification, and biomarker-guided intervention, rather than as a purely mechanistic phenomenon.

## Conclusions

Ferroptosis offers a coherent but still evolving pharmacological framework for understanding how inflammation, iron dysregulation, and lipid peroxidation contribute to CC. The strongest current evidence indicates that tumor-immune signals can transmit ferroptotic stress to host tissues, especially through neutrophil-derived LCN2 in lung CC and tumor-derived exosomal miR-203a-3p in pancreatic cancer-associated muscle wasting. These mechanisms support a causal role for ferroptosis in selected cachexia contexts, while underscoring the need to avoid overgeneralization from nonspecific oxidative signatures. The central translational insight is the therapeutic paradox: ferroptosis induction can benefit tumor control, yet host metabolic tissues may suffer collateral ferroptotic injury that accelerates muscle and adipose wasting. Future interventions should, therefore, pursue split-ferroptosis control-inducing ferroptosis where it is oncologically useful and preserving ferroptosis resistance where it protects host function.Tissue-resolved mechanisms, longitudinal biomarkers, functional endpoints, drug-delivery strategies, and careful integration with cancer therapy will be required. With these safeguards, ferroptosis-targeted strategies may help move cachexia management from nonspecific supportive care toward mechanism-guided host protection and therapeutic-index-based pharmacology. Future ferroptosis-based interventions in CC will require a tumor-host pharmacological perspective to balance selective tumor vulnerability with the preservation of systemic tissue integrity.

## Acknowledgments

This work was supported by the 10.13039/501100001809National Natural Science Foundation of China (grant no. 82103021) and the 10.13039/501100002858China Postdoctoral Science Foundation (grant no. 2022M721725), both awarded to S.D.; the Nantong First People’s Hospital Provincial and Ministerial-Level High-Level Science and Technology Project Cultivation Fund (grant no. YPYJJYB24015), awarded to Y.L.; the 14th Five-Year Plan Nantong Clinical Medical Center (Innovation Platform); and the Youth Medical Expert Training Program of the Nantong Municipal Health Commission. The figures were created using BioRender.com.

## Author contributions

Conceptualization, Y.L., S.D., and R.C.; writing – original draft, Y.L. and S.D.; writing – review & editing, Y.L., R.C., and S.D.; supervision, Y.L., S.D., and R.C.; funding acquisition, Y.L. and S.D.; visualization, R.C.; data curation, R.C.; validation, S.D.; formal analysis, S.D.

## Declaration of interests

The authors declare no conflict of interest.
